# Harnessing combination therapy: Current treatments, recent advancements, and future directions in Alzheimer's disease

**DOI:** 10.1016/j.tjpad.2025.100327

**Published:** 2025-10-27

**Authors:** Howard M Fillit, Jacques Touchon, Bruno Vellas, Laura K Nisenbaum, Aaron H Burstein

**Affiliations:** a Alzheimer’s Drug Discovery Foundation (ADDF), New York, USA; b University of Montpellier, Montpellier, France; c I.H.U Health Age, Toulouse University Hospital, INSERM CERPOP, University of Toulouse, France

## 1 Aging and the development of Alzheimer’s disease

Advances in modern medicine have increased the human lifespan, leading to more elderly people worldwide [[1], [2], [3]]. Based on data from the United Nations and World Bank, the global population of individuals aged ≥65 years was approximately 750 million in 2023. Notably, about 67-70 % of this population lives in developing countries, representing approximately 500 to 525 million older adults [4]. The United States Census Bureau estimated that over 55 million US citizens were over the age of 65 in 2020, making up almost 17 % of the US population [5]. As the population ages, the prevalence of age-related conditions (eg, type 2 diabetes, cardiovascular diseases (CVDs), cancers, and neurodegenerative disorders is expected to increase [2,3]. Alzheimer’s disease (AD) is among the top contributors to disability and mortality in the elderly, with an estimated 7.2 million US citizens age 65 and over living with AD dementia as of 2025 [6]. Without major medical innovations to prevent or cure AD, this number is projected to increase to 13.8 million by 2060 [6].

Aging is a universal process resulting in progressive and irreversible decline throughout the entire body [7]. Throughout life, organisms decline at molecular and cellular levels in response to various stressors including genomic instability, epigenetic alterations, loss of proteostasis, mitochondrial dysfunction, and cellular senescence [7]. In the brain, cerebral atrophy, white and grey matter degradation, and neuropathological protein accumulation (including amyloid-β [Aβ] plaques and tau tangles) gradually manifest throughout aging [[8], [9], [10], [11]]. These aging-associated pathological changes lead to the development of cognitive decline that may manifest as reduced processing speed, reasoning, episodic memory, and spatial visualization [12]. Moreover, aging-associated changes to the brain increase the risk for the development of AD [6,11]. Additional aging-related effects, including chronic inflammation, impaired autophagy, vascular dysfunction, and synaptic loss, contribute to the development and progression of AD development [1]. The recent and major advances in geroscience research will most probably unveil new targets. Loss of neurons in the cerebral cortex and hippocampus, accumulation of Aβ plaques, and neurofibrillary tau tangles are the hallmarks of AD, leading to neuronal dysfunction, impaired memory, and reduced cognitive function [7,13].

Based on growing understanding of the multifaceted dysfunctional biologic processes contributing to the development of AD, combination therapies targeting more than 1 of these interrelated causes may provide the greatest opportunity for slowing the progression of AD [14]. Combination therapy strategies targeting key pathways in a synergistic or additive manner may potentially improve memory, attention, and reasoning compared with monotherapy [15]. Although current combination or coadministration approaches do not halt the progression of AD, they may potentially offer an orthogonal approach to managing symptoms and delaying disease progression [16]. The current AD drug development pipeline includes approaches leveraging pharmacodynamic combinations, pharmacokinetic combinations, and combinations aimed at enhancing penetration across the blood brain barrier [17]. As of June 2025, there are at least 10 clinical trials actively evaluating combination therapies in AD (Table 1). With the regulatory approval of anti-amyloid monoclonal antibodies (mAbs) and their integration into clinical practice, it is anticipated that future trials of putative therapies will need to consider the opportunities and challenges associated with investigating coadministration to patients on a background therapy with mAbs [17].

**Table 1:** Ongoing and recent clinical trials investigating combination therapies in AD.

Trial	Phase	Population	Treatments	Status	Results
SToMP-AD [18] NCT04685590	2 Randomized Controlled Double-blind	Patients aged ≥60 years with amnestic mild cognitive impairment or early AD	Senolytic therapy (dasatinib + quercetin)vs placebo	Active/not recruiting	Primary completion projected: January 2028
Study 202[19] NCT06602258	2 Randomized Controlled Double-blind	Early AD	E2814 (anti-tau + anti-Aβ) + lecanemabvs placebo + lecanemab	Recruiting	Primary completion projected: August 2027
MET-FINGER[20] NCT05109169	2 Randomized Controlled Double-blind	At risk of dementia	Metformin + FINGER 2.0 lifestyle interventionvs self-guided intervention	Recruiting	Primary completion projected: June 2027
ADEPT-1[21] NCT05511363	3 Randomized Controlled Double-blind	Patients aged 55-90 with possible or probable AD and ≥2 month history of psychotic symptoms	KarXT (xanomeline + trospium chloride)vs placebo	Recruiting	Primary completion projected: October 2026
NCT04570644[22]	1/2 Randomized Open-label	AD	ALZT-OP1a + ALZT-OP1b	Completed in January 2021	
NCT01872598[23]	3 Randomized Controlled Double-blind	Patients aged ≥50 years with AD dementia	Masitinib + cholinesterase inhibitor (donepezil, rivastigmine or galantamine) and/or memantine vs placebo	Completed in December 2020	Dubois B et. al. *Alzheimer’s Res Ther* 2023
PEGASUS[24] NCT03533257	2 Randomized Controlled Double-blind	AD	Sodium Phenylbutyrate + Taurursodiolvs placebo	Completed on November 6, 2020	Arnold SE, et al. *Alzheimers Dement (N Y).* 2024;10(3):e12487
NCT06996730 [25]	2/3 Double-blind Placebo-controlled Double-dummy	Patients with PSEN1 E280A mutation and non-randomized, placebo-treated non-carriers from the same kindred Autosomal-dominant AD	Donanemab + RG6289	Not yet recruiting	Primary completion is projected for December 2030
NCT06602258 [19]	2 Placebo-controlled Double-blind Parallel group Dose-finding	Early AD	Lecanemab + E2814	Active/not recruiting	Primary completion is projected: July 2026
NCT06957418 [26]	2 Platform trial Randomized	Late preclinical or early prodromal AD	Tau-directed therapies, alone or in combination with donanemab	Not yet recruiting	Primary completion projected: August 2028

### 1.1 Current treatment landscape

Memantine (a N-methyl-D-aspartate receptor [NMDAR] antagonist) and the cholinesterase inhibitors (ChEIs) rivastigmine, galantamine, and donepezil have shown benefits in maintaining or improving cognitive function, at least temporarily [27]. One of the most studied combination therapies for AD is the concurrent use of memantine and ChEIs, which has demonstrated a decreased rate of cognitive decline and reduced severity of anticipated neurobehavioral symptoms (such as aggression) compared with ChEI monotherapy [28]. However, such combinations primarily address the symptoms of AD rather than addressing the underlying biology associated with the progression of the disease [[27], [28]].

More recently, 2 anti-Aβ mAbs (lecanemab-irmb [29] and donanemab-azbt [30] have received full FDA approval as treatments for AD, with additional approvals either granted or under consideration in other regions, including the EU, Japan, China, South Korea, Israel, Australia, Brazil, and Mexico [31,32]. While both mAbs are associated with approximately 30 % slowing of clinical decline and corresponding clearance of amyloid plaque [16,[33], [34], [35]], the need to target other biological process (eg, neuroinflammation, autophagy, mitochondrial or metabolic dysfunction), as well to augment the clinical efficacy, remains.

### 1.2 Combination strategies and novel treatment approaches in AD

Remarkable results with combination therapies for the treatment of several chronic diseases including neurological conditions, CVD, oncology indications, and metabolic disorders have been noted [33]. Because of the complex nature of AD, upcoming combination therapies may serve to address multiple core features, copathologies, and nonspecific symptoms [16]. Given the key roles of other aging-related processes in the pathogenesis of AD, there is a strong rationale for combining agents that address different mechanisms as a means of achieving a more comprehensive impact on the disease process. Current strategies for the management of chronic metabolic diseases like diabetes, a risk factor for the development of AD [36], include multimodal approaches, such as simultaneous lifestyle and pharmacological interventions [37]. Glucagon-like peptide-1 (GLP-1) receptor agonists, including semaglutide, are approved in combination with diet and exercise to improve glycemic control and to reduce the risk of cardiovascular and renal complications in individuals with type 2 diabetes [38]. GLP-1 has established roles in systemic inflammation, vascular health, and microglia/astrocyte homeostasis [39], making it an attractive therapeutic target in AD, with data for liraglutide in mild to moderate AD [40] supporting the ongoing Phase 3 evoke study of semaglutide in early AD. The pleiotropic effects of this class of agents provide a strong rationale for considering GLP-1 receptor agonists as potential components of combination regimens for AD [39]. Moreover, because lifestyle has an impact on metabolic disease management [37] as well as AD onset and progression [41], ongoing studies such as MET-FINGER are investigating the effects of the anti-hyperglycemic agent metformin and targeted lifestyle changes as a potential disease-modifying treatment in AD [42]. Recent research work on brain stimulation techniques could lead to novel therapeutic approaches in the field.

Drug repurposing provides an important opportunity to leverage the known safety and pharmacokinetic profiles of existing drugs previously developed for 1 indication and explore their efficacy for new indications [16]. For example, the FDA recently approved a combination therapy of xanomeline and trospium chloride for the treatment of schizophrenia, representing the first approved antipsychotic with a cholinergic mechanism of action [43]. Researchers have indicated this combination therapy may be effectively repurposed for AD, with an ongoing Phase 3 clinical trial assessing the efficacy of combining xanomeline and trospium chloride to treat the neuropsychiatric symptoms of AD (ADEPT-1; NCT05511363) [43]. Artificial intelligence (AI) models based on real-world data, animal studies, and drug databases may be leveraged to identify prospective therapeutics and repurpose drugs for AD [16].

### 1.3 A comprehensive perspective on combination therapy for AD: key considerations

This edition of The Journal of Prevention of Alzheimer’s Disease (JPAD) addresses contemporary issues in advancing the exploration of combination therapy for AD. The first article provides a foundation for the edition through an overview of various types of combination therapies for treatment of AD as well as potential challenges for consideration [44]. The field has been conducting combination and coadministration studies for decades, exploring putative symptomatic and/or disease-modifying therapies added to a background of stable standard of care (SOC) treatment with symptomatic agents (i.e., acetylcholinesterase inhibitors alone or with memantine). The recently approved anti-amyloid mAbs, with their effects on slowing the rate of decline and changing canonical biomarkers in AD, elicit new considerations for designing trials of combination regimens that incorporate mAbs. The second article provides a discussion of considerations specifically related to coadministration or combination therapy with mAbs, including clinical trial design, statistics, biomarkers, and operational implementation [45]. Reflecting the burgeoning use of AI in drug development, this edition also includes an article addressing the use of AI in the selection of putative combination therapies for nonclinical and/or clinical exploration [46]. Statistical considerations for combination therapy trials are addressed in 2 papers providing frequentist and Bayesian approaches for considerations in the design and conduct of combination therapy trials for AD [47,48].

The penultimate article in this edition focuses on the potential role for combination of lifestyle intervention with pharmacologic approaches as a multifaceted approach to treatment [49]. The final article addresses opportunities and challenges associated with the development of combination therapy with repurposed therapies [50].

### 1.4 A vision for the future of treatment for AD

While combination therapy approaches offer opportunities for significant improvement in patient outcomes, potential challenges exist to achieving their successful investigation in clinical research and implementation in clinical practice [14]. A key unanswered question relates to the optimal trial infrastructure and the potential role of different trial designs. Adaptive trial designs such as platform trials or response-adaptive randomization could offer flexibility to allocate participants to promising treatment arms in real time based on the data from the ongoing AD trials, similar to application in other areas (eg, oncology) [14]. Notwithstanding, the ongoing DIAN-TU, Alzheimer Tau, and MET FINGERS trials provide excellent opportunities to build upon the experience of these investigators in executing such studies [42,[51], [52], [53]]. Another salient question relates to the utility of AI and machine learning in drug development trials. Although the use of these tools has developed rapidly and is expanding, there are still challenges, such as a lack of large training sets and benchmark drug combination datasets in the AD field to train and validate classic machine learning or deep learning models before experimental or clinical testing [46]. Indeed, further development is still needed in this area prior to any successful implementation.

Increasing adoption of anti-amyloid mABs in clinical practice will present an opportunity for the exploration of combination therapy with novel therapies. In such scenarios, several key issues must be addressed to enable effective trial design. These include defining strategies for enrolling patients already receiving mABs and determining how best to assess the safety and efficacy of add-on therapies administered alongside existing therapies. Additional considerations involve the optimal timing of treatment for introducing a novel agent following mAB initiation, identifying determinants of treatment response, evaluating the impact on clinical and biomarker assessments, and thoroughly interrogating the safety and tolerability of combination regimens. Thus, the next phase of development of combination therapy for AD will require significant investments, painstakingly building a new body of evidence, new ways of thinking about the disease, and the flexibility to adapt approaches as we learn new information [14].

Beyond the scientific and medical rationale supporting combination therapy for AD, there are very important operational considerations that will determine the feasibility of its implementation in real-world practice. One such consideration is determining the optimal stage in drug development to evaluate combination therapy. A common strategy is to first establish a minimal profile (defined as the essential characteristics of a therapeutic agent such as efficacy, safety, target engagement) that justify its inclusion in a combination regimen. However, there is also an opportunity to explore de novo evaluation of agents specifically within the context of combination therapy, which may reveal synergistic effects not evident in monotherapy trials. Another key unanswered question relates to repurposed drugs (eg, off patent). While these drugs offer valuable opportunities to develop effective combination regimens, it remains a challenge to garner the financial support to move forward with these agents given the perceived lack of commercial opportunity. Additionally, while current research efforts primarily focus on therapeutic regimens that modulate AD symptoms or alter disease progression, there is a compelling opportunity to investigate the use of combination therapy for AD prevention, a critical area of unmet need. Finally, a key goal of current clinical trials is obtaining regulatory approval; however, there are other factors beyond clinical evidence and approval that impact the incorporation of a treatment regimen into clinical practice. The overarching regulatory environment is a critical consideration that directly impacts drug development and, because regulatory requirements can vary across different countries and regions, they have a direct and long-term impact on shaping the AD therapeutic landscape, associated costs, and patient access to treatment. While the unaddressed questions above are beyond the scope of this editorial, they are integral to advancing treatment and will determine the environment in which future therapies will be developed.

## Conflicts of interest

The authors declare the following financial interests/personal relationships which may be considered as potential competing interests:

HMF provided consultation to Alector, Inc, LifeWorX, and TheKey. HMF is a chairman, independent data management committee at Alector, Inc and a member of clinical advisory board at ProMis Neurosciences Inc and advisor at LifeWorX and TheKey. HMF is an observer and on board of directors at Therini Bio Inc and ADmit Therapeutics SL.

JT declares receipts of grants or contracts from ReGenlife, Anavex Life Sciences Corp and Ariana Pharmaceuticals and owns stock options for ReGenlife. JT provided consultation and received honoraria from ReGenlife for lectures, presentations, speakers’ bureaus, manuscript writing or educational events. JT participated in a Data safety Monitoring Board or Advisory Board for ReGenlife, Anavex Life Sciences Corp and Ariana Pharmaceuticals.

BV reports a relationship with the IHU HealthAge (Research National Agency, France 2030) Toulouse University Hospital and is an investigator in clinical trials sponsored by several industry partners. BV has served in the past 3 years as SAB member for Biogen Inc, Alzheon Inc, Norvo Nordisk Inc, Eli Lilly and Company, Eisai Inc, Roche, JNJ Ltd without personal compensation. BV is part of the Clinical Trials on Alzheimer’s Disease (CTAD) Organizing Committee and the Journal of Prevention of Alzheimer's Disease (JPAD) editorial board.

LKN and AHB have no conflicts to declare.

## Funding

All authors report that administrative support, article publishing charges, and writing assistance for this article were provided by Alzheimer’s Drug Discovery Foundation.

## CRediT authorship contribution statement

**Howard M Fillit:** Writing – review & editing. **Jacques Touchon:** Writing – review & editing. **Bruno Vellas:** Writing – review & editing. **Laura K Nisenbaum:** Conceptualization, Writing – review & editing. **Aaron H Burstein:** Conceptualization, Writing – review & editing.

## Declaration of competing interest

The authors declare the following financial interests/personal relationships which may be considered as potential competing interests:

Howard Fillit reports a relationship with Therini Bio Inc that includes: board of directors membership. Howard Fillit reports a relationship with Alector Inc that includes: chairman, Independent Data Management Committee membership. Howard Fillit reports a relationship with ProMIS Neurosciences Inc that includes: clinical advisory board membership. Howard Fillit reports a relationship with LifeWorx that includes: consulting or advisory and board membership. Howard Fillit reports a relationship with TheKey that includes: consulting or advisory and board membership. Howard Fillit reports a relationship with ADmit Therapeutics SL that includes: board membership.

Laura K Nisenbaum reports a relationship with Alzheimer’s Drug Discovery Foundation that includes: employment.

Aaron Burstein reports a relationship with Alzheimer’s Drug Discovery Foundation that includes: employment.

Bruno Vellas reports a relationship with Biogen Inc that includes: consulting or advisory without personal compensation. Bruno Vellas reports a relationship with Alzheon Inc that includes: consulting or advisory without personal compensation. Bruno Vellas reports a relationship with Novo Nordisk Inc that includes: consulting or advisory without personal compensation. Bruno Vellas reports a relationship with Eli Lilly and Company that includes: consulting or advisory without personal compensation. Bruno Vellas reports a relationship with Roche that includes: consulting or advisory without personal compensation. Bruno Vellas reports a relationship with Eisai Inc that includes: consulting or advisory without personal compensation. Bruno Vellas reports a relationship with JNJ Ltd that includes: consulting or advisory without personal compensation. Bruno Vellas reports a relationship with Clinical Trials on Alzheimer’s Disease (CTAD) Organizing Committee that includes: board membership. Bruno Vellas reports a relationship with IHU HealthAge (Research National Agency, France 2030) Toulouse University Hospital and is an investigator in clinical trials sponsored by several industry partners: funding grants. Bruno Vellas is part of the Clinical Trials on Alzheimer’s Disease (CTAD) Organizing Committee.

Jacques Touchon reports a relationship with REGEnLIFE that includes: consulting or advisory, equity or stocks, funding grants, and speaking and lecture fees. Jacques Touchon reports a relationship with Anavex Life Sciences Corp that includes: consulting or advisory and funding grants. Jacques Touchon reports a relationship with Ariana Pharmaceuticals that includes: consulting or advisory and funding grants.

Authors report writing assistance was provided by Alzheimer’s Drug Discovery Foundation.

Given his role as part of the JPAD editorial board, Bruno Vellas had no involvement in the peer review of this article and had no access to information regarding its peer review. Full responsibility for the editorial process for this article was delegated to another journal editor. If there are other authors, they declare that they have no known competing financial interests or personal relationships that could have appeared to influence the work reported in this paper.
